# Essential components and validation of multi-specialty robotic surgical training curricula: a systematic review

**DOI:** 10.1097/JS9.0000000000002284

**Published:** 2025-02-04

**Authors:** Josephine Walshaw, Michael G. Fadel, Matthew Boal, Marina Yiasemidou, Muhammed Elhadi, Francesca Pecchini, Francesco Maria Carrano, Lisa H Massey, Matyas Fehervari, Omar Khan, Stavros A. Antoniou, Felix Nickel, Silvana Perretta, Hans F. Fuchs, George B. Hanna, Nader K. Francis, Christos Kontovounisios

**Affiliations:** a Leeds Institute of Medical Research, St James’s University Hospital, University of Leeds, Leeds, United Kingdom; b Department of Surgery and Cancer, Imperial College London, London, United Kingdom; c The Griffin Institute, Northwick Park and St Mark’s Hospital, London, United Kingdom; d Department of Colorectal Surgery, The Royal London Hospital, Barts Health NHS Trust, London, United Kingdom; e Tripoli University Hospital, Tripoli, Libya; f Division of General Surgery, Emergency and New Technologies, Baggiovara General Hospital, Modena, Italy; g Department of Medical and Surgical Sciences and Translational Medicine, Faculty of Medicine and Psychology, St Andrea Hospital, Sapienza University, Rome, Italy; h Department of Colorectal Surgery, Nottingham University Hospitals NHS Trust, Nottingham, United Kingdom; i Department of Bariatric Surgery, Maidstone and Tunbridge Wells NHS Trust, Kent, United Kingdom; j Population Sciences Department, St George’s University of London, London, United Kingdom; k Department of Bariatric Surgery, St George’s Hospital, London, United Kingdom; l Department of Surgery, Papageorgiou General Hospital, Thessaloniki, Greece; m Department of General, Visceral and Thoracic Surgery, University Medical Center Hamburg-Eppendorf, Hamburg, Germany; n IRCAD, Research Institute Against Digestive Cancer, Strasbourg, France, NHC University Hospital, Strasbourg, France; o Department of General, Visceral, Cancer and Transplantation Surgery, University Hospital Cologne, Cologne, Germany; p Department of Colorectal Surgery, Chelsea and Westminster Hospital NHS Foundation Trust, London, United Kingdom; q Department of Colorectal Surgery, Royal Marsden NHS Foundation Trust, London, United Kingdom; r 2nd Surgical Department, Evaggelismos Athens General Hospital, Athens, Greece

**Keywords:** robotic curriculum, robotic training, surgical curriculum, surgical training

## Abstract

**Introduction:**

The rapid adoption of robotic surgical systems has overtook the development of standardized training and competency assessment for surgeons, resulting in an unmet educational need in this field. This systematic review aims to identify the essential components and evaluate the validity of current robotic training curricula across all surgical specialties.

**Methods:**

A systematic search of MEDLINE, EMBASE, Emcare, and CINAHL databases was conducted to identify the studies reporting on multi-specialty or specialty-specific surgical robotic training curricula, between January 2000 and January 2024. We extracted the data according to Kirkpatrick’s curriculum evaluation model and Messick’s concept of validity. The quality of studies was assessed using the Medical Education Research Study Quality Instrument (MERSQI).

**Results:**

From the 3687 studies retrieved, 66 articles were included. The majority of studies were single-center (*n* = 52, 78.8%) and observational (*n* = 58, 87.9%) in nature. The most commonly reported curriculum components include didactic teaching (*n* = 48, 72.7%), dry laboratory skills (*n* = 46, 69.7%), and virtual reality (VR) simulation (*n* = 44, 66.7%). Curriculum assessment methods varied, including direct observation (*n* = 44, 66.7%), video assessment (*n* = 26, 39.4%), and self-assessment (6.1%). Objective outcome measures were used in 44 studies (66.7%). None of the studies were fully evaluated according to Kirkpatrick’s model, and five studies (7.6%) were fully evaluated according to Messick’s framework. The studies were generally found to have moderate methodological quality with a median MERSQI of 11.

**Conclusions:**

Essential components in robotic training curricula identified were didactic teaching, dry laboratory skills, and VR simulation. However, variability in assessment methods used and notable gaps in curricula validation remain evident. This highlights the need for standardized evidence-based development, evaluation, and reporting of robotic curricula to ensure the effective and safe adoption of robotic surgical systems.

## Introduction

The rapid advancement and integration of robotic systems into surgical practice has transformed the surgical landscape globally^[1]^. Robotic-assisted surgery (RAS) requires distinct skills that differ from those required for open and laparoscopic surgery, introducing a three-dimensional visual output and a higher degree of movement freedom^[2]^. Developing these specialized skills requires comprehensive training through a dedicated and structured program encompassing essential knowledge, safety principles, and technical proficiencies to achieve optimal surgical outcomes.

Surgical practice was significantly affected by the coronavirus (COVID-19) pandemic, which highlighted the need for a shift in surgical training approaches^[3,4]^. As healthcare systems navigate the post-pandemic landscape, it is essential to create resilient, accessible, and adaptable training programs that can withstand future challenges and ensure the advancement of surgical education, including RAS. However, there is a considerable variability in RAS training methods, including didactic approaches with lectures and theoretical learning, hands-on training with virtual reality (VR) simulation, and both dry and wet laboratory exercises^[5,6]^. Additionally, bedside training plays a crucial role in developing transferable skills, allowing surgeons to smoothly transition from the classroom to the operating room. Hands-on training may include aspects, such as patient positioning, procedure-specific port placement, and robot docking. The development of non-technical skills, such as communication, decision-making, and teamwork, is also equally important in ensuring comprehensive RAS competence^[5,7]^.

Despite the growing use of RAS, current training frequently relies on industry-led courses and independent fellowships, often lacking a formal, proficiency-based curriculum and validated accreditation process^[8,9]^. In addition, traditional training programs for robotic surgery only address specialists; there is a lack of validated programs that address the needs of surgical residents. Although some groups have developed advanced RAS training programs^[10–12]^, there is still a growing need for a standardized basic RAS training curriculum^[13]^. Such a curriculum would provide a consistent foundation in RAS, ensuring that all practitioners reach a minimum level of competence before progressing to more complex procedures. Evaluating the effectiveness of such training programs requires a comprehensive approach using standardized frameworks to ensure that a training curriculum can positively impact clinical practice.

This systematic review aims to analyze the essential components, assessment methods, evaluation, and validity of existing curricula across single and multi-specialty RAS training programs. This can identify gaps in current training approaches and provide a foundational reference for the development of robust RAS training programs, ensuring that surgeons are effectively trained to deliver optimal patient outcomes using robotic platforms.

## Methods

### Protocol and registration

The review was prospectively registered in the PROSPERO database (registration ID CRD42023418429) and the protocol, as a part of the development of a consensus in robotic training for gastrointestinal (GI) surgical trainees, which was published by the European Robotic Surgery Consensus (ERSC) study group^[14]^. This process follows five stages: (i) the formation of a steering committee, (ii) a systematic review of the existing multi-specialty robotic surgical curricula, (iii) a pan-European survey to capture current robotic training practices, potential challenges, and opportunities for improvement, (iv) a Delphi process to achieve agreement on crucial aspects of a robotic training curriculum, and (v) a dissemination strategy.

This systematic review was performed to identify and define the essential components of RAS training curriculum across all surgical specialties in line with the Cochrane Handbook for Systematic Reviews of Interventions^[15]^. This review has been reported following the Preferred Reporting Items for Systematic Reviews and Meta-Analyses (PRISMA) 2020 guidelines^[16]^ (Supplementary material S1, http://links.lww.com/JS9/D877) and AMSTAR – Assessing the Methodological Quality of Systematic Reviews – Guidelines^[17]^.

### Eligibility criteria

All studies reporting on multi-specialty or specialty-specific RAS training curricula – including single procedure curricula – in any surgical specialty for novice or expert surgeons were eligible for inclusion. Randomized controlled trials (RCTs), prospective or retrospective observational studies were included. The exclusion criteria includes: (i) laparoscopic or other non-RAS curriculum, (ii) using robotics or simulation to train in non-RAS procedures, (iii) curriculum for medical students and non-surgeons, (iv) case reports, editorials, reviews, expert opinions, and conference abstracts without available full text, and (v) non-English language articles.

### Information sources

A comprehensive search was conducted on studies published between January 2000 and January 2024 using MEDLINE, EMBASE, Emcare, and CINAHL databases. The reference lists of all included studies and screened full texts were hand-searched for additional relevant papers. When full texts were not obtainable via conventional access methods, the authors and publishing journals were approached to request the full article text.

### Search strategy

The search strategy was formulated for each database using relevant Medical Subject Headings (MeSH) terms, which included robotic surgery, robot-assisted surgery, training, simulation, syllabus, curriculum, and education. The full search strategy is available in Supplementary material S2, http://links.lww.com/JS9/D877.

### Selection and data collection process

Search results were uploaded onto the Covidence systematic review software^[18]^, and duplicates were removed. Two independent reviewers screened titles and abstracts against the inclusion and exclusion criteria, and full text of potentially relevant articles for inclusion. Two independent reviewers performed the data extraction using a bespoke data extraction spreadsheet (Microsoft Excel Version 2408)^[19]^. Any disagreement between reviewers was resolved through consensus or a third reviewer.

### Data items

Extracted data include: (i) study characteristics, including year of publication, country, study design, sample size, and specialty; (ii) curriculum components – e.g., didactic component, VR simulation, live case observation, dry laboratory skills, wet laboratory skills, bedside assistance training, dual console training, proctored clinical training, and non-technical skills training; and (iii) assessment methods – e.g., direct assessment, video assessment, and objective outcome tools used, if applicable). We also extracted data according to Kirkpatrick’s model of curriculum evaluation^[20]^ and Messick’s concept of validity^[21]^ (Table 1).

**Table 1:** Description and examples of the components of Kirkpatrick’s Model of Curriculum Evaluation and Messick’s Concept of Validity. Adapted from^[20,21,46]^

Component	Description	Examples
Kirkpatrick’s Model of Curriculum Evaluation		
Reaction	Measures participants’ immediate satisfaction and engagement with the curriculum	Participants complete surveys after the curriculum, indicating their satisfaction with materials and teaching
Learning	Assesses the knowledge or skills acquired from the curriculum, can be objective or subjective	Pre- and post-tests measure participants’ performance of a skill before and after the curriculum
Behaviour	Evaluates the application of learned skills	Evaluation of participants applying newly learned skills in their job – e.g., in person, survey
Results	Measures the long-term impact of the curriculum	Tracking participants’ performance after completing the curriculum – e.g., operative performance – or reassessing performance – skill retention
Messick’s Concept of Validity		
Content	Establishes the usefulness and realism of the curriculum – face validity – and if the curriculum content reflects the intended goals – content validity	Content validity – Curriculum has been developed by experts – e.g., Delphi consensus
		Face validity – Participants feedback for usefulness and realism
Response process	Analyzes how well raters respond to the curriculum and evaluates the steps taken to enhance validity	Training raters to evaluate consistently, randomly assigning participants to different raters, blinding information from raters – e.g., participant identity – and using expert raters to ensure accurate evaluation
		Virtual reality simulator automated performance metrics eliminate rater bias in assessments
Internal structure	Measures how well the domains of the curriculum or assessment align with the underlying construct	Internal consistency – Curriculum or assessment consistently measure the same construct
		Intra-rater reliability – Same rater gives consistent scores for the same performance
		Inter-rater reliability – Agreement between different raters scores for the same performance
		Virtual reality simulator automated performance metrics eliminate rater subjectivity in assessments
Relationship to other variables	Examines the relationship between the curriculum and other variables of interest	Concurrent validity – Outcomes of the curriculum are consistent with other established methods
		Construct validity – Whether experts consistently outperform novices and distinguish levels of expertise
		Predictive validity – Curriculum performance predicts real-world performance
		Learning curve – In a proficiency-based progression model, participants show a consistent improvement over time
Consequences	Evidence of the impact of the curriculum or assessment	Established thresholds for passing, failing, or achieving certain performance levels
		Impact of curriculum on participants’ learning – e.g., surgeon’s confidence, operative outcomes

### Synthesis methods

Extracted data items were tabulated and a narrative synthesis approach was conducted in line with the Guidance on the Conduct of Narrative Synthesis in Systematic Reviews^[22]^. Continuous data were presented as medians and interquartile ranges (IQR), and categorical data were presented as frequencies and percentages. We used Kirkpatrick’s model of curriculum evaluation to assess how well the curriculum achieved its desired educational outcomes. The following four aspects were considered: (i) reaction – measure of participants’ reaction, e.g., feedback; (ii) learning – measure of participants’ learning, measured objectively or subjectively; (iii) behavior – applying training to work; and (iv) results – long-term impact on participants’ learning/outcomes, e.g., skill retention assessment, and predictive validity improving operative performance. We also used Messick’s concept of validity to ensure the quality and validity of curricula and assessment tools, confirming that they accurately measure participant performance and provide reliable results. The following five aspects were assessed in the curriculum design, implementation, and assessment for each study: (i) content – face and content validity; (ii) response process – analysis of raters; (iii) internal structure – reliability analyses; (iv) relationship to other variables – concurrent, construct, or predictive validity and learning curve; and (v) consequences – impact of assessment or curricula.

### Quality assessment

Quality assessment was performed by two independent reviewers and disagreements were resolved by a third reviewer. The validated Medical Education Research Study Quality Instrument (MERSQI)^[23]^ was used to assess the study quality out of a total 18 points, taking into account the study design, sampling, type of data, the validity of assessment instruments, data analysis, and study outcomes.

## Results

**Figure 1.**
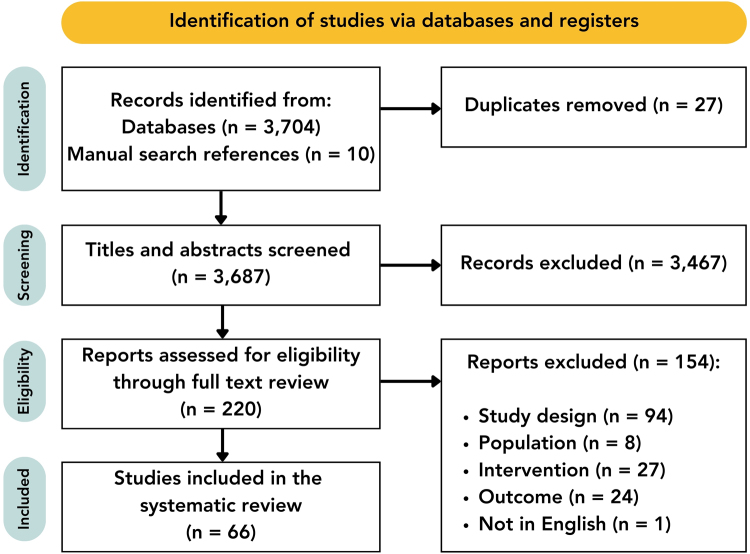
The flowchart shows the literature search and study selection process according to PRISMA guidelines.

A total of 3677 articles were identified from databases for title and abstract screening after duplication removal, and 10 additional studies identified through hand-searching references. Of the 3687 abstracts that were screened, 3467 were excluded. After reviewing the full texts of 220 studies, 154 were excluded based on the inclusion and exclusion criteria, resulting in 66 studies being included for the final review (Fig. 1).

### Study characteristics

The 66 included studies comprised of five RCTs (7.6%), and 58 observational studies (87.9%), out of which 15 were retrospective (22.7%) and 43 were prospective (65.2%) in nature. There were three articles (4.5%) containing curriculum development descriptions. The majority of studies were conducted in a single-center (*n* = 52, 78.8%), with 14 multicenter studies (21.2%). Among the multicentre studies, the median number of centers was four (IQR 2.5–8), and two studies did not specify the number of centers involved. Most studies were conducted in the USA (*n* = 45, 68.2%), with four multinational studies (6.1%). The individual study characteristics are summarized in Table 2.

**Table 2:** Study characteristics

First author	Year	Country	Study design	Number of centers	Number and type of participants	Robotic platform	Surgical specialty	Primary focus of study	MERSQI score
Ahmad, *et al*^[30]^	2021	USA	Prospective Observational	4	30 surgeons (oncology fellows)	da Vinci (Intuitive Surgical, Sunnyvale, CA)	Surgical Oncology	Implement robotic surgery curriculum using virtual reality and inanimate reality drills	12
Al Abbas, *et al*^[61]^	2021	USA	Retrospective Observational	1	187 patients and 4 surgeons + unclear number of fellows	Not stated	Hepatobiliary Surgery	Evaluate the impact of surgeon mentorship and a formal curriculum on mitigating the learning curve for robotic distal pancreatectomy in subsequent generations of surgeons	11.5
Ali, *et al*^[62]^	2007	USA	Prospective Observational	1	30 patients; Unclear number of surgeons (minimally invasive surgery fellow)	Zeus (Intuitive Surgical, Sunnyvale, CA)	Upper Gastrointestinal Surgery	Demonstrate that incremental increase in surgical responsibility would be effective in teaching surgical trainees to perform complex robotic surgical tasks in a safe and efficient manner	10.5
Alrasheed, *et al*^[63]^	2014	USA	Prospective Observational	1	10 surgeons (plastic surgeons at various levels of training)	da Vinci (Intuitive Surgical, Sunnyvale, CA)	Plastic Surgery	Develop a validated assessment instrument and assess the learning curve for robotic microsurgery	11
Alterio, *et al*^[64]^	2023	USA	Prospective Observational	1	3 surgeons (robotically trained)	da Vinci (Intuitive Surgical, Sunnyvale, CA)	General Surgery	Identify drills that represented the skills needed for actual robotic operations, and which would be well-accepted by learners	4.5
Aradaib, *et al*^[65]^	2019	Ireland	Prospective Observational	1	55 patients and 4 surgeons (laparoscopic colorectal surgeons)	da Vinci (Intuitive Surgical, Sunnyvale, CA)	Colorectal Surgery	Describe early institutional experience with adoption of robotic colorectal surgery using structured training	9.5
Arain, *et al*^[32]^	2012	USA	Prospective Observational	1	55 surgeons (various levels of training)	da Vinci (Intuitive Surgical, Sunnyvale, CA)	Multi-specialty	Assess metric reliability, investigate the feasibility of implementing the curriculum, and evaluate educational benefits	12
Ayabe, *et al*^[66]^	2018	USA	Retrospective Observational	1	98 patients; Unclear number of surgeons (junior and senior residents)	da Vinci (Intuitive Surgical, Sunnyvale, CA)	General Surgery	Whether robotic cholecystectomy can safely be introduced to junior residents	9.5
Bell, *et al*^[67]^	2015	Australia	Retrospective Observational	1	48 patients and 4 surgeons (laparoscopic colorectal surgeons)	da Vinci (Intuitive Surgical, Sunnyvale, CA)	Colorectal Surgery	Describe the process taken to establish a robotic colorectal surgery programme in a large academic private hospital	8.5
Beulens, *et al*^[68]^	2021	Netherlands	Prospective Observational	Multiple (Not stated number)	29 surgeons (various levels of training)	da Vinci (Intuitive Surgical, Sunnyvale, CA)	Urology	Analyze the effects on surgical skills by the introduction of an advanced course in robotic-assisted surgery for residents	11.5
Butterworth, *et al*^[69]^	2021	USA	Prospective Observational	1	17 surgeons (various levels of training)	Versius (Cambridge Medical Robotics Surgical, Cambridge, UK)	Multi-specialty	Evaluate the effectiveness of the Versius training programme	12
Dioun, *et al*^[70]^	2017	USA	Prospective Observational	1	24 surgeons (various levels of training)	da Vinci (Intuitive Surgical, Sunnyvale, CA)	Gynecology	Develop and evaluate the robotic simulation curriculum	10
Dulan, *et al*^[71]^	2012	USA	Prospective Observational	1	2 surgeons (novice and expert)	da Vinci (Intuitive Surgical, Sunnyvale, CA)	Multi-specialty	Develop a proficiency-based robotic curriculum	8
Fastenberg, *et al*^[72]^	2018	USA	Prospective Observational	1	20 surgeons (residents with no/minimal robotic experience)	da Vinci (Intuitive Surgical, Sunnyvale, CA)	Otolaryngology	Develop an introductory resident transoral robotic surgery curriculum	8.5
Kingma, *et al*^[73]^	2020	Germany	Prospective Observational	1	70 operations	da Vinci (Intuitive Surgical, Sunnyvale, CA)	Esophagogastric Surgery	Demonstrate the effectiveness of a structured training pathway in robot-assisted minimally invasive esophagectomy	9
					1 surgeon (expert)				
Foell, *et al*^[74]^	2013	Canada	Prospective Observational	1	37 surgeons (various levels of training)	da Vinci (Intuitive Surgical, Sunnyvale, CA)	Multi-specialty	Develop a simulation-based robotic surgery basic skills curriculum	9
Formisano, *et al*^[75]^	2019	Italy	Prospective Observational	1	2 surgeons (junior attendings competent in basic minimally invasive surgery)	da Vinci (Intuitive Surgical, Sunnyvale, CA)	Colorectal Surgery	Assess the efficacy of an institutional training program for robotic right hemicolectomy	3.5
Fujimura, *et al*^[76]^	2016	Japan	Prospective Observational	1	242 operations and 8 surgeons (robotic novices)	da Vinci (Intuitive Surgical, Sunnyvale, CA)	Urology	Development and evaluation of a mentored robot-assisted radical prostatectomy training pathway	6.5
Galloway, *et al*^[28]^	2012	USA	Retrospective Observational	1	24 surgeons (residents)	da Vinci (Intuitive Surgical, Sunnyvale, CA)	Gynecology	Incorporate robotic surgery training into the standard four-year gynaecology curriculum	5.5
Geller, *et al*^[29]^	2011	USA	Prospective Observational	1	17 surgeons (residents)	da Vinci (Intuitive Surgical, Sunnyvale, CA)	Gynecology	Establish a standardized robotic surgical training programme	4.5
Gerull, *et al*^[77]^	2020	USA	Prospective Observational	1	31 surgeons (residents)	da Vinci (Intuitive Surgical, Sunnyvale, CA)	Multi-specialty	Assess the impact of a virtual reality robotic curriculum on operative performance	9
Gleason, *et al*^[9]^	2022	USA	Prospective Observational	1	53 surgeons (trainees)	da Vinci (Intuitive Surgical, Sunnyvale, CA)	Multi-specialty	Assess the impact of a virtual reality robotic curriculum on post-test scores	10
Gomez, *et al*^[78]^	2015	USA	Prospective Observational	1	22 surgeons (novice robotic surgeons)	da Vinci (Intuitive Surgical, Sunnyvale, CA)	General Surgery	Develop and evaluate a proficiency-based virtual reality curriculum	11
Grannan, *et al*^[25]^	2021	USA	Retrospective Observational	1	161 operations and 43 surgeons (residents)	da Vinci (Intuitive Surgical, Sunnyvale, CA)	General Surgery	Pilot a robotic general surgery resident training curriculum	4.5
Green, *et al*^[26]^	2021	USA	Curriculum development description	8	N/A	da Vinci (Intuitive Surgical, Sunnyvale, CA)	General Surgery	Develop a robotic general surgery resident training curriculum	5
Hogg, *et al*^[33]^	2017	USA	Prospective Observational	1	17 surgeons (various levels of training)	da Vinci (Intuitive Surgical, Sunnyvale, CA)	Hepatobiliary Surgery	Evaluate the validity of a virtual reality curriculum	11
Hung, *et al*^[34]^	2017	USA	Prospective Observational	1	21 surgeons (robotic trainees at various levels of training)	Not stated	Urology	Evaluate the structured learning and proficiency assessment in robotic surgery	13
Hung, *et al*^[79]^	2013	USA	Prospective Observational	2	49 (38 trainees, 11 experts)	da Vinci (Intuitive Surgical, Sunnyvale, CA)	Urology	Assessing the validity of three robotic training methods	14
Kajiwara, *et al*^[80]^	2011	Japan	Curriculum development description	1	2 surgeons (expert thoracic surgeons)	da Vinci (Intuitive Surgical, Sunnyvale, CA)	Thoracic Surgery	Determine the feasibility of using the training system for educating thoracic surgeons in robotic surgery for lung cancer	12
Kiely, *et al*^[81]^	2015	Canada	Randomized controlled trial	1	27 surgeons (various levels of training)	da Vinci (Intuitive Surgical, Sunnyvale, CA)	Multi-specialty	Whether a proficiency-based, virtual reality robotic suturing curriculum improves the robotic suturing performance	13.5
Kim, *et al*^[24]^	2023	USA	Prospective Observational	3	25 surgeons (various levels of training)	da Vinci (Intuitive Surgical, Sunnyvale, CA)	Multi-specialty	Assess whether trainees demonstrate improvement in a standardized knot-tying task after completing a virtual reality robotic curriculum	13
Klompmaker, *et al*^[82]^	2021	USA	Retrospective Observational	1	237 patients; Unclear number of surgeons (experience with open hepatobiliary surgery and residents)	da Vinci (Intuitive Surgical, Sunnyvale, CA)	Hepatobiliary Surgery	Train practicing surgeons in robot-assisted distal pancreatectomy and assess the impact on five domains of healthcare quality	13
Larcher, *et al*^[31]^	2019	Multiple	Retrospective Observational	1	200 patients 1 surgeon (trainee) Delphi: 27 experts	da Vinci (Intuitive Surgical, Sunnyvale, CA)	Urology	Define and validate a curriculum for robot-assisted partial nephrectomy	11.5
Madureira, *et al*^[83]^	2017	Brazil	Retrospective Observational	1	293 operations; Unclear number of surgeons (general and colorectal surgeons)	da Vinci (Intuitive Surgical, Sunnyvale, CA)	General Surgery	Describe the implementation of a training program in robotic surgery and point the General Surgery procedures that can be performed with advantages using the robotic platform.	10
Mark Knab, *et al*^[84]^	2018	USA	Retrospective Observational	1	30 surgeons (oncology and hepatobiliary fellows)	da Vinci (Intuitive Surgical, Sunnyvale, CA)	Surgical Oncology	Evolution and outcomes of a proficiency-based robotic training program for surgical oncology fellows	10
Martin, *et al*^[85]^	2021	USA	Prospective Observational	2	20 surgeons (residents)	RobotiX Mentor (3D Systems, Cleveland, OH)	Multi-specialty	Determine if robotic surgery novices demonstrate improved technical skill performance after completing the Fundamentals of Robotic Surgery training	12.5
Mehrabi, *et al*^[86]^	2006	Germany	Prospective Observational	1	4 surgeons (trainees)	da Vinci (Intuitive Surgical, Sunnyvale, CA)	Multi-specialty	Design a clear, standardized, and reproducible training method that can qualitatively and quantitatively evaluate the surgical performance when a surgical robotic system is introduced.	12.5
Melnyk, *et al*^[87]^	2022	USA	Prospective Observational	1	5 teams (each with a surgeon, first assistant, and circulating nurse)	da Vinci (Intuitive Surgical, Sunnyvale, CA)	Urology	Design and implement a simulation-based curriculum for training interdisciplinary robotic surgical teams in emergency robotic undocking protocols and open conversion	12.5
Merriman, *et al*^[88]^	2023	USA	Retrospective Observational	1	24 surgeons (gynaecology residents)	da Vinci (Intuitive Surgical, Sunnyvale, CA)	Gynecology	Describe and evaluate a two-phase robotic curriculum for obstetrics and gynecological residents	13
Moit, *et al*^[27]^	2019	USA	Retrospective Observational	1	6 surgeons (general surgery residents)	da Vinci (Intuitive Surgical, Sunnyvale, CA)	General Surgery	Evaluate a standardized robotic training curriculum designed to improve robotic surgical skills among general surgery residents	13
Moles, *et al*^[89]^	2009	USA	Prospective Observational	1	7 surgeons (otolaryngology residents)	da Vinci (Intuitive Surgical, Sunnyvale, CA)	Otolaryngology	Developing a training program for teaching robotic skills to residents	12
Mü ller, *et al*^[90]^	2023	Germany	Prospective Observational	1	154 patients and 2 surgeons (experts)	da Vinci (Intuitive Surgical, Sunnyvale, CA)	General Surgery	Evaluate and help understand the pathway to reach surgical expert levels using a profciency-based approach introducing RAMIE at a high-volume center	9
Mustafa, *et al*^[91]^	2019	USA	Retrospective Observational	1	4 patients before curriculum, 99 patients after curriculum, 164 patients long-term, and Unclear number of surgeons (residents)	Not stated	General Surgery	Examine if the implementation of a robotics curriculum enhances minimally invasive surgical training	8.5
Panteleimonitis, *et al*^[92]^	2021	Multiple	Prospective Observational	26	35 surgeons (various levels of training)	da Vinci (Intuitive Surgical, Sunnyvale, CA)	Colorectal Surgery	Examine the short-term outcomes of a structured training programme for robotic colorectal surgery in an international setting	13.5
Panteleimonitis, *et al*^[93]^	2018	Multiple	Prospective Observational	2	82 patients and 3 surgeons (experienced minimally invasive surgeons, no robotic experience)	da Vinci (Intuitive Surgical, Sunnyvale, CA)	Colorectal Surgery	Exhibit the feasibility and safety of a training pathway	13.5
Puliatti, *et al*^[35]^	2021	Belgium	Prospective Observational	1	19 surgeons (experts and novices) Delphi: 13 experts	da Vinci (Intuitive Surgical, Sunnyvale, CA)	Urology	Development and validation of performance metrics for robotic procedures	12.5
Radi, *et al*^[94]^	2022	USA	Prospective Observational	1	41 surgeons (residents)	da Vinci (Intuitive Surgical, Sunnyvale, CA)	Multi-specialty	Evaluate the feasibility, effectiveness, and transferability of a mastery-based curriculum using a new virtual reality robotic simulator for surgery resident training	13
Ramirez Barriga, *et al*^[95]^	2022	USA	Curriculum development description	1	N/A	da Vinci (Intuitive Surgical, Sunnyvale, CA)	General Surgery	Provide standardized training to ensure robotic surgical experience before sitting at the console in the operating room	11.5
Raza, *et al*^[96]^	2014	USA	Prospective Observational	3	61 surgeons (experts and novices)	da Vinci (Intuitive Surgical, Sunnyvale, CA)	General Surgery	Report the ability of a simulation-based robotic training curriculum to assess and distinguish between different performance levels of operator experience	14.5
Rice, *et al*^[97]^	2020	USA	Prospective Observational	1	514 operations 4 surgeons (robotic novices)	Not stated	Hepatobiliary Surgery	Evaluate the association of mentorship and a formal proficiency-based skills curriculum with the learning curves of 3 generations of surgeons and the association with increased patient safety	10
Richmon, *et al*^[98]^	2011	USA	Prospective Observational	1	20 patients and 1 surgeon (Otolaryngology surgeon)	da Vinci (Intuitive Surgical, Sunnyvale, CA)	Otolaryngology	Review strategy and experiences by implementing a TORS program	5.5
Rusch, *et al*^[99]^	2018	Multiple	Prospective Observational	4	4 surgeons (gynaecology surgeons, robotic novices)	da Vinci (Intuitive Surgical, Sunnyvale, CA)	Gynecology	Experiences of the Society of European Robotic Gynaecology Surgery pilot curriculum in terms of feasibility, effectiveness, and the potential for certification	13.5
Satava, *et al*^[100]^	2020	USA	Randomized controlled trial	12	99 surgeons (various levels of training)	da Vinci (Intuitive Surgical, Sunnyvale, CA)	Multi-specialty	Compare the Fundamentals of Robotic Surgery curriculum with current training paradigms	14
Scott, *et al*^[101]^	2020	Denmark	Prospective Observational	1	22 surgeons (novices and experts)	RobotiX Mentor (3D Systems, Cleveland, OH)	Multi-specialty	Design and collect the validity evidence for a cross‐specialty basic robotic surgical skills training program using the Robotix Mentor virtual reality simulator	11
Shay, *et al*^[102]^	2019	USA	Prospective Observational	1	20 surgeons (residents)	da Vinci (Intuitive Surgical, Sunnyvale, CA)	Otolaryngology	Assess short and long-term retention of robotic skills	11
Stegemann, *et al*^[103]^	2013	USA	Randomized controlled trial	3	53 surgeons (various levels of training)	da Vinci (Intuitive Surgical, Sunnyvale, CA)	Multi-specialty	Develop and establish the effectiveness of a simulation-based robotic curriculum	15
Tarr, *et al*^[104]^	2014	USA	Randomized controlled trial	8	165 surgeons (residents)	Not stated	Multi-specialty	Determine if the robotic dry lab curriculum improved basic robotic skills	12
Thomas, *et al*^[105]^	2021	UK	Retrospective Observational	1	90 operations and 2 surgeons (colorectal consultants)	da Vinci (Intuitive Surgical, Sunnyvale, CA)	Colorectal Surgery	Experience of implementation and adaptation of a structured robotic colorectal program	13
Turbati, *et al*^[106]^	2023	USA	Prospective Observational	1	18 surgeons (novices and experts)	da Vinci (Intuitive Surgical, Sunnyvale, CA)	Multi-specialty	Validate the SimNow resident robotic basic simulation curriculum	10.5
Unruh, *et al*^[107]^	2023	USA	Retrospective Observational	1	681 operations and 25 surgeons (colorectal residents)	da Vinci (Intuitive Surgical, Sunnyvale, CA)	Colorectal Surgery	Describe the components of the curriculum and characterize the immediate impact of implementation	6
Valdis, *et al*^[108]^	2015	Canada	Randomised controlled trial	1	20 surgeons (trainees)	da Vinci (Intuitive Surgical, Sunnyvale, CA)	Cardiac Surgery	Evaluate the impact of a virtual reality simulation curriculum to improve the robotic cardiac surgery skill acquisition	13.5
Volpe, *et al*^[109]^	2015	Belgium	Prospective Observational	Multiple (Not stated number)	10 surgeons (fellows)	da Vinci (Intuitive Surgical, Sunnyvale, CA)	Urology	Establish the feasibility, acceptability, face validity, and educational impact of a structured training curriculum for robot-assisted radical prostatectomy	13.5
White, *et al*^[110]^	2018	USA	Prospective Observational	1	5 surgeons (senior otolaryngology residents)	da Vinci (Intuitive Surgical, Sunnyvale, CA)	Otolaryngology	Develop a multi-faceted curriculum to prepare the residents for transoral robotic surgery and to assess the effectiveness of a curriculum	7
Wiener, *et al*^[111]^	2015	USA	Retrospective Observational	1	16 surgeons (junior and senior residents)	da Vinci (Intuitive Surgical, Sunnyvale, CA)	Urology	Define the time needed by urology residents to attain proficiency in robotic surgery to refine the curriculum	10.5
Winder, *et al*^[112]^	2016	USA	Prospective Observational	1	20 surgeons (residents)	da Vinci (Intuitive Surgical, Sunnyvale, CA)	General Surgery	Implementation of a robotic surgical curriculum in general surgery	4
Zwart, *et al*^[113]^	2022	Netherlands	Prospective Observational	7	275 operations 15 surgeons (trained hepatobiliary surgeons)	da Vinci (Intuitive Surgical, Sunnyvale, CA)	Hepatobiliary Surgery	Assess the feasibility and safety of a multicentre training program in robotic pancreatoduodenectomy	13.5

MERSQI, Medical Education Research Study Quality Instrument; RAMIE, Robotic-Assisted Minimally Invasive Esophagectomy; TORS, Trans-Oral Robotic Surgery, N/A, not available

### Curriculum overview

Sixteen studies (24.2%) focused on multispecialty curricula, with the most common specialty-specific curricula being General Surgery (*n* = 12, 18.2%), Urology (*n* = 9, 13.6%), and Colorectal Surgery (*n* = 7, 10.6%). The participant grade varied, with 33 studies involving only trainees/residents (50.0%), 12 studies including only consultants/attending/staff (18.2%), and 21 studies having mixed groups of participants (31.8%).

The da Vinci system (Intuitive Surgical, Sunnyvale, CA) was the most commonly used robotic platform (*n* = 57, 86.4%), with other platforms, including the RobotiX Mentor (3D Systems, Cleveland, OH) (*n* = 2, 3.0%), Versius (Cambridge Medical Robotics Surgical, Cambridge, UK) (*n* = 1, 1.5%), and Zeus (Intuitive Surgical, Sunnyvale, CA) (*n* = 1, 1.5%). Curriculum length was reported in 28 studies (42.4%), with the shortest programme lasting 5 hours and 37 minutes for a VR curriculum^[24]^, and the longest spanning 5 years as a part of a residency training program^[25–27]^.

Curriculum cost analysis was provided in five studies (7.6%). One study reported simulation equipment costs at $1000^[28]^, with another study noting individual training modules ranging between $25 and $122, with an annual cost of $178 to replace the disposable dissection and suturing models^[29]^. The cost of individual training instruments was noted, with a large needle driver costing $1650, the round-tip scissors at $1465, and the ProGrasp forceps at $1650, each having approximately 30 uses^[29]^. Additional cost considerations included video recording materials, such as GoPro cameras with accessories ($600 each), online video hosting platforms ($400 per year), editing software ($600 per year), and crowdsourced video graders ($22.27 per video)^[30]^. The reported expenditure associated with each live operating case included in one curriculum was estimated to be €1920^[31]^.

### Curriculum components

**Figure 2.**
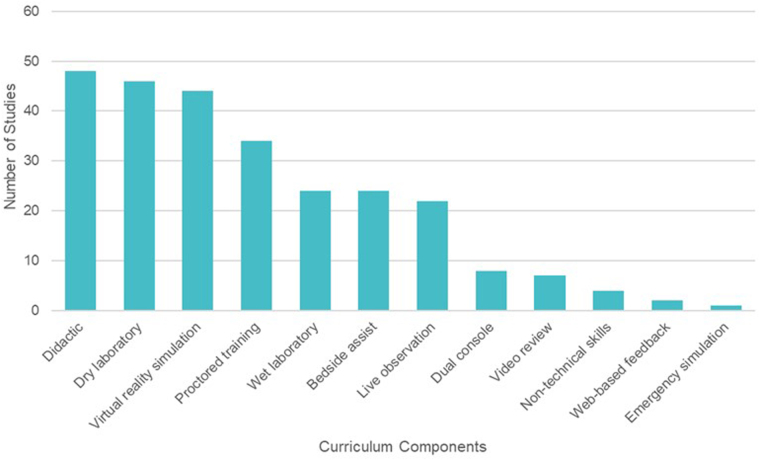
Components of robotic surgical training curricula reported in studies included in this systematic review.

The curriculum components varied across the studies, with no single curriculum component being universally adopted (Fig. 2). The most commonly included components were didactic training (*n* = 48, 72.7%), dry laboratory skills (*n* = 46, 69.7%), and VR simulation (*n* = 44, 66.7%). Wet laboratory skills were encompassed in 24 studies (36.4%) – 11 animal alone, four cadaveric alone, six animal and cadaveric, two high-fidelity artificial organs, and one unclear tissue type. Dedicated bedside assistance training was included in 24 studies (36.4%). Live operating experience within the robotic curriculum was incorporated through observation of live cases (*n* = 22, 33.3%), dual console training (*n* = 8, 12.1%), and proctored clinical training (*n* = 34, 51.5%). Non-technical skills in robotic surgery were included in four curricula (6.1%). Additional components that were noted were the review of robotic videos (*n* = 7, 10.6%), web-based feedback (*n* = 2, 3.0%), and emergency undocking simulation (*n* = 1, 1.5%).

### Assessment methods

The methods used to assess learning included both direct observation (*n* = 44, 66.7%) and video assessment (*n* = 26, 39.4%). Objective outcome measures were applied in 44 studies (66.7%), with VR/simulation metrics (*n* = 15, 22.7%), the Global Evaluation Assessment of Robotic Skills (GEARS) (*n* = 13, 19.7%), and the Objective Structured Assessment of Technical Skills (OSATS) (*n* = 8, 12.1%) being some of the most commonly applied measures (Table 3). Self-assessment methods were reported in four studies (6.1%), while four other studies (6.1%) did not use any form of assessment.

**Table 3:** Assessment metrics used in robotic curricula

Assessment Metric	Description	Number of studies utilised (%)
Simulator/Virtual Reality Metrics	Quantitative measure of performance in virtual reality simulators on various aspects of the task being performed.	15 (22.7%)^[9,25,33,68-70,72,74,78,91,94,96,102,106,111]^
Global Evaluation Assessment of Robotic Skills (GEARS)	Measurement of six dimensions for robotic-assisted surgery technical skill evaluation, including depth perception, bimanual dexterity, efficiency, force sensitivity, autonomy, and robotic control.	13 (19.7%)^[24,27,34,69,79,81,85,88,99,100,107-109]^
Task-specific metrics	Metrics used to assess specific aspects of performance during a task, such as the time taken to complete a procedure or the number of errors made.	10 (15.2%)^[28,29,33,71,74,84,85,87,89,103]^
Objective Structured Assessment of Technical Skills (OSATS)	Evaluates a trainee’s performance using standardized criteria, including tissue handling, economy of movement, handling and knowledge of instruments, use of assistants, flow and forward planning of the operation, and knowledge of specific procedures.	8 (12.1%)^[24,30,33,84,94,95,99,104]^
Cumulative Sum Control Chart (CUSUM)	Statistical tool used to monitor a trainee’s performance over time, detecting trends and changes in their proficiency.	5 (7.6%)^[73,82,90,92,93]^
Goal Assessment Score (GAS)	Scoring for robotic colorectal surgery assessment, including robotic docking, colonic dissection, total mesorectal excision, and resection and anastomosis.	4 (6.1%)^[65,92,93,105]^
Procedure-specific scoring system	Assessment of proficiency/technical ability specific to an operation(s) – e.g., prostatectomy – or part of an operation – e.g., docking – being performed	4 (6.1%)^[26,34,73,109]^
Modified Fundamentals of Laparoscopic Surgery (FLS)	Program designed to teach and evaluate the knowledge, judgment, and skills fundamental to laparoscopic surgery	2 (3.0%)^[32,71]^
Non-Technical Skills for Surgeons (NOTSS) Rating System	Evaluates five categories of non-technical skills for safe surgical practice, including situation awareness, decision-making, task management, communication & teamwork, and leadership.	2 (3.0%)^[88,99]^
Task Load Index (TLX)	Self-assessment of the cognitive load experienced during a procedure, including mental demand, physical demand, temporal demand, performance, effort, and frustration.	2 (3.0%)^[77,87]^
Global Operative Assessment of Laparoscopic Skills (GOALS)	Rating scale for laparoscopic skills, including depth perception, bimanual dexterity, efficiency, tissue handling, and autonomy.	1 (1.5%)^[81]^
Structured assessment of robotic microsurgical skills	Combines the Structured Assessment of Microsurgical Skills scoring system – dexterity, visuospatial ability, and operative flow – with robotic skills – camera movement, depth perception, wrist articulation, atraumatic needle handling, and atraumatic tissue handling – and overall performance	1 (1.5%)^[63]^
Robotic Ottowa Surgical Competency Operating Room Evaluation (RO-SCORE)	Robotic modification of the O-SCORE tool, including overall technical performance – camera control, energy control, needle control, tissue handling, instrument control – efficiency and flow, and communication	1 (1.5%)^[77]^
Electrodermal activity	Non-invasive measure of electrical properties of skin to assess stress levels during tasks	1 (1.5%)^[87]^

### Curriculum validation

**Figure 3.**
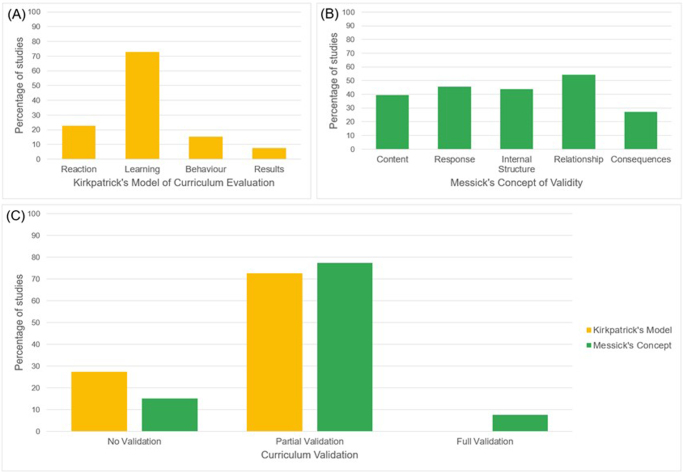
Curricula validation; (A) percentage of studies meeting the components for Kirkpatrick’s model of curriculum evaluation, (B) percentage of studies meeting the components for Messick’s concept of validity, and (C) percentage of studies having no, partial, or full validation.

According to Kirkpatrick’s model of curriculum evaluation, none of the included studies were fully validated. Forty-eight (72.7%) studies were partially validated: (i) reaction (*n* = 15, 22.7%), (ii) learning (*n* = 48, 72.7%), (iii) behavior (*n* = 10, 15.2%), and (iv) results (*n* = 5, 7.6%). Eighteen (27.3%) studies were not validated for any of the Kirkpatrick’s criteria points. For Messick’s concept of validity, five (7.6%) curricula were fully validated. Fifty-one (77.3%) studies were partially validated: (i) content (*n* = 26, 39.4%), (ii) response process (*n* = 30, 45.5%), (iii) internal structure (*n* = 29, 43.9%), (iv) relationship to other variables (*n* = 36, 54.5%), and (v) consequences (*n* = 18, 27.3%). Ten (15.2%) study curricula were not validated for any of Messick’s concepts of validity (Fig. 3). In general, the included studies had a moderate methodological quality with a median MERSQI score of 11 (IQR 9–13) out of 18 points.

## Discussion

This systematic review provides a comprehensive overview of the existing RAS training curricula across multiple specialties globally, highlighting the diversity in curriculum components, assessment strategies, and validation. The most commonly reported curricula components in the literature include didactic learning, dry laboratory skills, and VR simulation. While most curricula were found to be partially validated for both Kirkpatrick’s and Messick’s frameworks, five curricula achieved full validation according to Messick’s concept of validity^[9,32–35]^. These efforts demonstrate progress in developing effective RAS training programmes, though broader validation across additional curricula is still needed. The review findings underscore significant variability and a lack of standardization, emphasizing the need for a more unified and validated approach to RAS training.

### Variability in curriculum design

An important finding from this review is the variation in RAS curricular components. Didactic training, dry laboratory skills, and VR simulation were the most commonly included elements, appearing in over two-thirds of the reviewed studies. Wet laboratory training also helps surgeons adapt to the lack of haptic feedback in RAS by focusing on visual cues for tissue handling^[5]^. This component plays a vital role in bridging the gap between theoretical learning and the operating room; however, it was only included in 36.4% of RAS curricula. The inclusion of proctored training in more than half the curricula emphasizes the importance of hands-on, practical experience in preparing surgeons for real-world applications. However, bedside assistance and dual console trainings were less frequently incorporated.

The 2006 SAGES-MIRA – Society of Gastrointestinal and Endoscopic Surgeons and Minimally Invasive Robotic Association – Consensus on RAS training emphasized the need for combining didactic courses, hands-on training, and guided operating room components for comprehensive robotic surgery education^[36]^. However, no single component was universally adopted across all curricula in this review, reflecting the lack of standardized RAS training in the current literature.

The relatively low inclusion of non-technical skills training suggests a further gap in research in this area, despite the critical role these skills play in effective teamwork, communication, and decision-making in the operating room^[37,38]^. A recent systematic review highlighted a paucity of non-technical skills reporting in RAS, with only three bespoke objective assessment tools being used^[39]^. This underscores the need for greater integration and assessment of non-technical skills in RAS training programs to ensure that surgical teams are equipped to handle the challenges of the operating room environment.

Transparency in reporting costs associated with RAS training was also found to be limited, with insufficient reporting of both participant and site expenses. Detailed cost reporting is essential for evaluating the financial feasibility and resource allocation of training programs and better informed decision-making in curriculum development to ensure that these programs are both cost-effective and accessible.

### Assessment methods

The assessment methods used in RAS training were equally varied, incorporating both direct observation and video assessments, along with a range of objective outcome measures. Direct observation was the most frequent form of assessment used, reflecting a preference for real-time evaluation techniques. A systematic review by Grüter *et al*^[40]^ suggested that validated video-based objective surgical quality assessment tools enable objective assessment of surgical performance; however, current surgical video recording practices are heterogeneous^[41]^. Beyond assessment, recording procedures offer the additional benefit of identifying errors, allowing surgeons to learn from these instances and fostering a culture of continuous improvement in performance and patient safety^[42]^. Self-assessment can be a valuable tool for fostering reflective practice and lifelong learning, yet it remains underutilized in surgical training^[43,44]^. There is often a poor correlation between self-assessment and independent evaluation^[45]^; therefore, it should be used as a complementary tool to enhance personal insight rather than as a replacement for expert feedback.

Despite the use of objective measures in two-thirds of the studies, variability in scoring metrics raises questions about the consistency and reliability of skill assessment across different programs. A systematic review by Boal *et al*^[46]^ similarly highlighted the significant variability in the approach and evaluation of tools for RAS assessment, with only a few having undergone robust validation. Although these tools offer great potential for objectively evaluating surgical skills, further evaluation is required before they are integrated into accreditation processes.

### Challenges in curriculum validation

The IDEAL – Idea, Development, Exploration, Assessment, and Long-term monitoring – framework for surgical robotics advocates for the evaluation of novel training methods using validated frameworks, such as Messick’s, and emphasizes the need for standardized RAS training programmes overseen by independent accrediting bodies^[13]^. While many studies achieved partial validation according to Kirkpatrick’s model of curriculum evaluation and Messick’s concept of validity, there are notable shortcomings in the current curricula.

None of the curricula achieved full validation under Kirkpatrick’s model, with most focusing on the “Learning” component while neglecting other crucial aspects, like behavioral changes and long-term impact. Similarly, validation according to Messick’s framework was also limited, with only a small percentage of curricula being fully validated. These omissions may have significant implications for the quality of training and its translation into improved clinical practice. Effective curricula should not only assess immediate learning outcomes, but also consider the broader impact on surgical practice and patient care. The moderate methodological quality, as reflected by the median MERSQI score, further emphasizes the need for higher-quality research in this area.

RAS training curricula should be aimed to be evaluated with the same rigour as the National Training Programme for Laparoscopic Colorectal Surgery (Lapco), which successfully implemented a structured, competency-based approach to training laparoscopic colorectal surgeons in England^[47]^. The use of one-to-one expert supervision, objective assessment tools, and a standardized, high-quality framework for training and progression demonstrated how new surgical techniques can safely and effectively be integrated into practice^[48–51]^.

Since the completion of the review, multiple emerging curricula have been published. Although these curricula fall outside the search range, the authors have provided a summary and evaluation of their frameworks. The Association of Laparoscopic Surgeons of Great Britain and Ireland (ALSGBI) have developed an accreditation-based programme for pre-clinical core robotic skills^[52]^. This curriculum successfully fulfilled all five Messick’s validity domains, and two of the four Kirkpatrick’s domains. The curriculum achieved a MERSQI score of 14, and is the first curriculum, to our knowledge, to implement Objective Clinical Human Reliability Analysis (OCHRA) error analysis tool. The Robotic Surgery Training Curriculum (RoSTraC) is a further standardized program, specifically designed for surgical residents^[53]^. This curriculum evaluation fulfilled four of the five Messick’s domains, and three of the four Kirkpatrick’s domains, demonstrating a MERSQI score of 13.5.

Additionally, the European Society of Coloproctology (ESCP) “ColoRobotica” pathway is a structured colorectal robotic training pathway, which recently published a guideline including statements on the knowledge, technical, and non-technical skills; assessment of competency; and credentialing for robotic colorectal surgery^[54]^. It is a comprehensive curriculum covering key modules of a robotic training pathway from e-learning and simulation training to live case observation and proctorship, with proficiency-based progression and accreditation through objective assessment (MERSQI 17). Although there are no published studies evaluating its curriculum, the ESCP society have published on a robotic-specific Training The Trainer pathway^[55]^ and a validated robotic low anterior resection proficiency-based metrics objective assessment tool^[56]^. This provided evidence across all five domains of the Messick’s concept, although benchmarking has not been defined.

### Limitations

It is important to acknowledge the limitations that may affect the interpretation of this systematic review’s findings. The included studies exhibited variability, making it challenging to draw definitive conclusions or directly compare results across studies to identify best practices. Most studies were performed at a single centre and were observational in nature, further limiting the generalizability of the results. The majority of studies also focused on curricula performed on the da Vinci system, the most commonly used robotic platform worldwide^[57]^; however, an increasing number of alternative robotic platforms are becoming available^[58]^. This narrow focus in the literature on curricula performed on a single platform may not fully capture the breadth of training experiences across different robotic systems. As new platforms emerge, it is crucial to include them in research to ensure that the training curricula remain relevant and effective across various technologies. Ultimately, further research is required to design and evaluate a training curriculum that can be universal across several platforms.

### Implications for future research and practice

The results of this review highlight several key areas for future research and practice. First, there is a clear need to develop and implement standardized RAS training curricula that incorporate best practices from across the field. Such curricula should be based on a thorough understanding of the essential components of RAS training and must be validated using rigorous, comprehensive evaluation frameworks. Second, the assessment methods used in RAS training should be standardized to ensure consistency and reliability across programmes. This includes a greater emphasis on the objective measures, such as simulation metrics and validated assessment tools, as well as the incorporation of self-assessment and potential integration of AI through automated skills assessments and advanced intraoperative metrics^[59]^. Finally, future studies should aim for higher methodological rigor and transparency, ensuring that their findings can be confidently applied to practice. There is a need for more research on the long-term impact of RAS training on clinical outcomes, as well as the cost-effectiveness of different training methods. Additional work should be society- and clinician-led, in collaboration with industries.

Aligning with the review findings, our recent pan-European survey identifies the critical need for a unified curriculum to address the gaps in training, assessment, and certification^[60]^. The survey further emphasizes the importance of integrating early simulation training, dual console learning, bedside assisting, and robust assessment tools. Together, these insights will guide the subsequent Delphi processes to develop a European Robotic Surgery Consensus for a robotic training curriculum used for GI trainees^[14]^. This should lead to the development of a formal curriculum for robotic training for GI trainees, featuring essential curriculum components, assessment tools, and minimum requirements for certification.

## Conclusions

This systematic review highlights the essential components reported in RAS training curricula. It was also evident that there is a significant variability and lack of standardization in RAS training assessment methods, and validation processes. While progress has been made in developing and implementing RAS training programs, there is still a further need for a more unified approach that ensures all surgeons receive high-quality and effective training. By addressing the gaps identified in this review, the field of RAS can move towards a more consistent, reliable, and impactful training programs that ultimately improve surgical outcomes and patient care.

## Data Availability

All data generated or analyzed during this study are included in this published article.

## Research registration unique identifying number (UIN)

The study protocol was registered on the Open Science Framework (https://osf.io/br87d/) and this systematic review was registered in the PROSPERO database (registration ID CRD42023418429).
